# Mitigation of tobacco bacteria wilt with microbial degradation of phenolic allelochemicals

**DOI:** 10.1038/s41598-022-25142-0

**Published:** 2022-12-01

**Authors:** Xiaohan Chang, Yi Wang, Jingguo Sun, Haibo Xiang, Yong Yang, Shouwen Chen, Jun Yu, Chunlei Yang

**Affiliations:** 1 State Key Laboratory of Biocatalysis and Enzyme Engineering, School of Life Science, Hubei University, Wuhan, 430062 China; 2 Tobacco Research Institute of Hubei Province, Wuhan, 430030 China

**Keywords:** Plant sciences, Natural hazards

## Abstract

Long-term continuous monoculture cropping of tobacco leads to high incidence of tobacco bacterial wilt (TBW) caused by *Ralstonia solanacearum*, which threatening world tobacco production and causing great economy loss. In this study, a safe and effective way to control TBW by microbial degradation of phenolic allelochemicals (PAs) was explored. Eleven kinds of PAs were identified from continuous tobacco cropping soil. These PAs exhibited various effects on the growth, chemotaxis and biofilm formation of *R*. *solanacearum*. Then we isolated eight strains of *Bacillus*, one strain of *Brucella,* one strain of *Enterobacter* and one strain of *Stenotrophomonas* capable of degrading these PAs. The results of degradation assay showed that these isolated strains could degrade PAs both in culture solutions and soil. Besides, the incidence of TBW caused by *R*. *solanacearum* and deteriorated by PAs were significantly decreased by treating with these degrading strains. Furthermore, six out of eleven isolated strains were combined to degrade all the identified PAs and ultimately sharply reduced the incidence of TBW by 61.44% in pot experiment. In addition, the combined degrading bacteria could promote the plant growth and defense response. This study will provide a promising strategy for TBW control in tobacco production.

## Introduction

Tobacco bacterial wilt (TBW) is a serious soil-borne bacterial disease caused by *Ralstonia solanacearum*, which occurs widely in the tobacco-producing areas of China and becomes a worldwide problem^1^. *R. solanacearum* can infect not only the tobacco, but also hundreds of crops in more than 50 families including eggplant, tomato and potato^2^. Once the disease occurs, it will seriously affect the growth and development of tobacco, the quality of tobacco leaves, and even cause the death of the whole plant. It has a great impact on the yield and quality of tobacco and has become a restricting factor for the development of tobacco production^1^.

Recent studies showed the involvement of allelochemicals in the incidence of TBW^3–6^. Allelochemicals are secondary metabolites released from plants or microorganisms into the surrounding environment, which exert positive or negative effects on the growth and development of the nearby plants or microorganisms in an interspecific or intraspecific form^3^. Many studies indicated that some allelochemicals could inhibit plant growth through interfering with photosynthesis, respiration, membrane transport, seed germination and root tissue growth, causing serious ecological and economic problems, such as poor crop yield and continuous cropping obstacles^3^. Besides, they can promote the incidence of TBW. On the one hand, allelochemicals can be served as the carbon source to promote the growth of *R. solanacearum*, which badly infect many kinds of crops*.* In addition, some allelochemicals can stimulate or induce pathogenic colonization to host plant roots and the formation of biofilm, which eventually initiate bacterial wilt and cause severe economic loss^4^. For example, cinnamic acid, fumaric acid and oxalic acid secreted from tobacco roots could significantly induce the colonization of *R. solanacearum* in tobacco roots, and benzoic acid and phenylpropionic acid intensively stimulated the reproduction of *R. solanacearum* in rhizosphere soil^4–6^. Therefore, how to reduce these harmful allelochemicals is critical to the control of TBW.

Phenolic compounds are common allelochemicals among many plants, including tobacco^7^, rice^8^, peanut^9^, strawberry^10^, melon^11^, etc. The main harmful allelochemicals are phenolic acids and phenolic acid esters, such as coumaric acid^12^, benzoic acid (BA)^13^, *p*-hydroxybenzoic acid (PHBA)^13^, vanillic acid (VA)^14^ and p-hydroxybenzaldehyde (POBA)^15^. There are many microorganisms that can degrade allelochemicals in nature and it is a safe and environmentally friendly way to use these microorganisms to reduce the harmful allelochemicals. A lot of phenolic allelochemical-degrading bacteria and fungi have been reported. Caffeic acid, ferulic acid, *p*-coumaric acid, BA, and PHBA can be degraded by *Aspergillus niger*^16^ *Bacillus subtilis*^17^, *Acinetobacter* and *Klebsiella oxytoca* FZ-8^18^. However, there are few reports on microorganisms degrading a wide range of phenolic allelochemicals (PAs) at the same time to biocontrol towards TBW. In this study, we firstly identified eleven kinds of PAs from tobacco rhizosphere soil and evaluated their stimulating and inducing effects on *R*. *solanacearum*. Then eleven strains capable of degrading these PAs were successfully isolated and their degrading efficiencies were characterized. At last, the biocontrol capacity of TBW with these strains were analyzed in pot experiment.

## Results

### Identification and quantification of rhizosphere soil phenolic allelochemicals (PAs)

A total of eleven kinds of PAs, including phthalic acid (PHA), p-hydroxybenzoic acid (PHBA), benzoic acid (BA), vanillic acid (VA), p-hydroxybenzaldehyde (POBA), vanillin (VN), diisobutyl phthalate (DIBP), 2,4-Di-tert-butylphenol (DTBP), di-n-butyl phthalate (DBP), diisooctyl phthalate (DIOP) and terephthalic acid (PTA), were identified through GC–MS analysis and quantitatively analyzed by internal standard method. PHA, DTBP and PTA were not detected in non-continuous cropping soil (CCS0) and CCS1 soil, while their relative contents increased with the continuous cropping year last, and the highest relative content reached 0.53%, 0.2% and 0.12% respectively in CCS10 soil (Table 1). The other PAs also accumulated with the increased year of continuous cropping, and their relative contents in CCS10 soil were 4- to 40-fold higher than those in CCS0 soil.

**Table 1:** The relative contents of rhizosphere phenolic allelochemicals (PAs) in tobacco-planting soils with different continuous cropping years.

Rhizosphere PAs	Relative contents (%)				
	CCS0	CCS1	CCS2	CCS4	CCS10
DTBP	0.00 ± 0.00c	0.01 ± 0.007c	0.05 ± 0.014bc	0.14 ± 0.054a	0.16 ± 0.066a
DIBP	0.00 ± 0.00b	0.00 ± 0.00b	0.01 ± 0.006b	0.02 ± 0.011b	0.53 ± 0.135a
DIOP	0.02 ± 0.003b	0.05 ± 0.023b	0.03 ± 0.015b	0.06 ± 0.035b	0.24 ± 0.066a
DBP	0.03 ± 0.004c	0.11 ± 0.027bc	0.12 ± 0.03abc	0.17 ± 0.065ab	0.22 ± 0.068a
BA	0.01 ± 0.003b	0.05 ± 0.015ab	0.07 ± 0.035ab	0.14 ± 0.052a	0.12 ± 0.064a
POBA	0.002 ± 0.0007b	0.01 ± 0.003b	0.03 ± 0.015b	0.07 ± 0.023a	0.08 ± 0.032a
PTA	0.01 ± 0.004b	0.07 ± 0.026b	0.08 ± 0.021b	0.1 ± 0.035b	0.21 ± 0.076a
PHA	0.00 ± 0.00b	0.00 ± 0.00b	0.02 ± 0.008b	0.04 ± 0.019b	0.2 ± 0.065a
VA	0.08 ± 0.015c	0.23 ± 0.061bc	0.3 ± 0.106b	0.37 ± 0.102ab	0.52 ± 0.109a
VN	0.11 ± 0.031b	0.32 ± 0.083ab	0.28 ± 0.055ab	0.27 ± 0.05a	0.43 ± 0.144a
PHBA	0.00 ± 0.00b	0.00 ± 0.00b	0.05 ± 0.01ab	0.08 ± 0.02a	0.12 ± 0.037a

*DTBP, 2,4-Di-tert-butylphenol; DIBP, diisobutyl phthalate; DIOP, diisooctyl phthalate; DBP, di-n-butyl phthalate; BA, benzoic acid; POBA, p-hydroxybenzaldehyde; PTA, terephthalic acid; PHA, phthalic acid; VA, vanillic acid; VN, vanillin; PHBA, p-hydroxybenzoic acid; CCS0, non-continuous cropping soils; CCS1, CCS2, CCS4 and CCS10, continuous cropping soils where only tobacco was planted for 1, 2, 4, and 10 years respectively. Different letters indicate significant difference (*P* < 0.05) according to Duncan’s multiple range test.

### Growth conditions of R. solanacearum 1–1 in different kinds of rhizosphere phenolic allelochemicals (PAs)

To determine whether the identified PAs contributed to the incidence of TBW, the effects of PAs at different concentrations on the growth of *R. solanacearum* 1–1 were evaluated. As shown in Table 2, DIBP and DBP promoted the growth of *R. solanacearum* 1–1 under all tested concentrations, and the highest values of OD_600_ reached 6.50 and 5.50 at the concentration of 2 mg/L and 4 mg/L respectively. BA, PTA, PHBA, VN, DTBP and DIOP promoted the growth of *R. solanacearum* 1–1 at low concentration while showed inhibition effects at high concentration, in which the optimum concentration of DTBP and DIOP was 1 mg/L and 2 mg/L respectively. BA, PTA, PHBA and VN had slight stimulation effect on *R. solanacearum* 1–1 growth at the concentration of 0.1 mg/L while the OD_600_ values decreased sharply when the concentration of BA reached 0.5 mg/ml and beyond. In addition, the values of OD_600_ declined when PHA, VA or POBA was added to the culture medium under all tested concentrations, suggesting that these phenolic allelochemicals exhibited antagonistic effect on the growth of *R. solanacearum* 1–1.

**Table 2:** The effects of different concentrations of rhizosphere phenolic allelochemicals (PAs) on the growth of *R. solanacearum* 1–1 (OD_600_).

Rhizosphere PAs	*R. solanacearum* 1–1 (OD_600_)					
	0 mg/L	0.1 mg/L	0.5 mg/L	1 mg/L	2 mg/L	4 mg/L
BA	3.73 ± 0.14a	3.97 ± 0.45a	2.04 ± 0.23b	1.65 ± 0.12b	1.18 ± 0.19c	0.68 ± 0.12d
PTA	3.73 ± 0.14ab	4.16 ± 0.32a	3.25 ± 0.29bc	3.03 ± 0.37 cd	3.15 ± 0.24c	2.58 ± 0.34d
PHA	3.73 ± 0.14a	2.58 ± 0.31b	2.50 ± 0.23b	2.19 ± 0.27b	1.33 ± 0.32c	0.89 ± 0.17c
PHBA	3.73 ± 0.14ab	3.98 ± 0.23a	3.47 ± 0.34b	2.12 ± 0.28c	1.43 ± 0.17d	0.74 ± 0.22e
VA	3.73 ± 0.14a	1.85 ± 0.16b	1.26 ± 0.22c	1.04 ± 0.15 cd	0.83 ± 0.16d	0.34 ± 0.15e
VN	3.73 ± 0.14a	3.86 ± 0.27a	2.40 ± 0.22b	2.16 ± 0.25b	1.39 ± 0.15c	0.86 ± 0.09d
POBA	3.73 ± 0.14a	3.59 ± 0.19ab	3.29 ± 0.21b	2.78 ± 0.21c	1.75 ± 0.21d	0.72 ± 0.13e
DTBP	3.73 ± 0.14b	3.71 ± 0.38b	3.86 ± 0.29ab	4.34 ± 0.30a	2.19 ± 0.35c	1.00 ± 0.18d
DBP	3.73 ± 0.14c	3.92 ± 0.38c	3.93 ± 0.32c	4.33 ± 0.31bc	4.60 ± 0.37b	5.50 ± 0.37a
DIBP	3.73 ± 0.14c	4.10 ± 0.33c	5.77 ± 0.40b	6.03 ± 0.35ab	6.50 ± 0.49a	5.43 ± 0.34b
DIOP	3.73 ± 0.14bc	4.09 ± 0.24b	5.22 ± 0.31a	5.53 ± 0.43a	3.92 ± 0.35b	3.30 ± 0.12c

*Different letters indicate significant difference (*P* < 0.05) according to Duncan’s multiple range test.

### Assessment of the chemotactic reaction of R. solanacearum 1–1 in response to PAs

**Figure 1:**
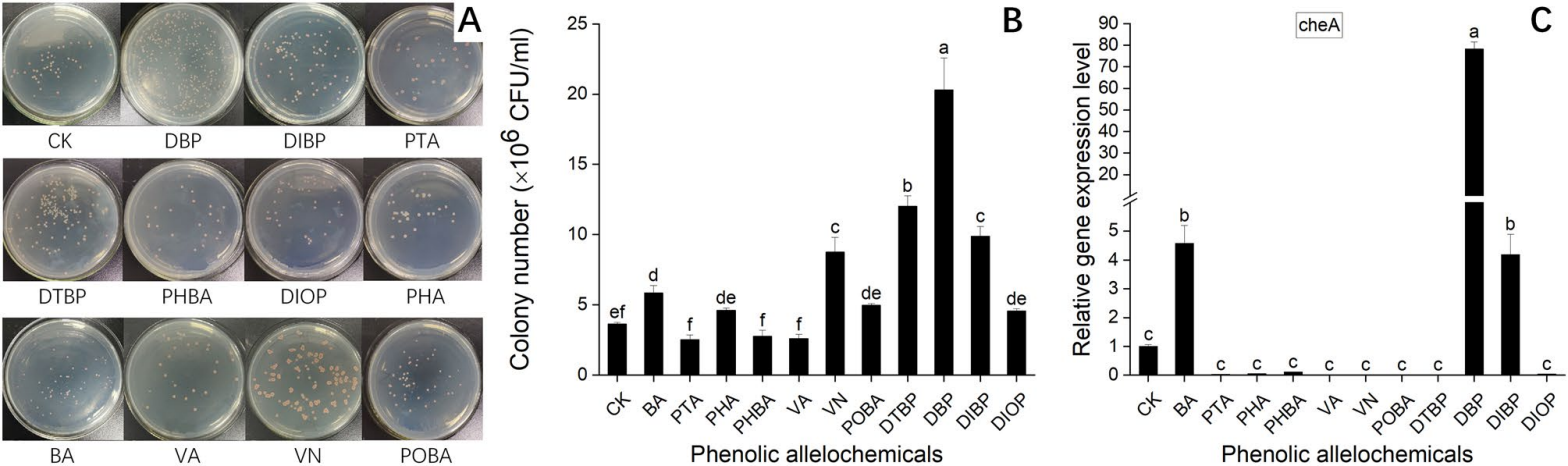
Chemotactic effect of *Ralstonia solanacearum* 1–1 to phenolic allelochemicals (PAs). (**A**), chemotactic response of *Ralstonia solanacearum* 1–1 to eleven PAs; (**B**), the bar chart was plotted with the data obtained from (**A**); (**C**), the relative expression level of *cheA* gene of *Ralstonia solanacearum* 1–1 in NB medium supplemented with PAs. BA, benzoic acid; PTA, terephthalic acid; PHA, phthalic acid; PHBA, p-hydroxybenzoic acid; VA, vanillic acid; VN, vanillin; POBA, p-hydroxybenzaldehyde; DTBP, 2,4-Di-tert-butylphenol; DBP, di-n-butyl phthalate; DIBP, diisobutyl phthalate; DIOP, diisooctyl phthalate.

To further determine whether the PAs had other effects on *R. solanacearum*, the chemotaxis assay was performed. Our results showed that *R. solanacearum* 1–1 could be significantly attracted by DBP, DTBP, DIBP, VN and BA, and the populations of *R. solanacearum* were as high as 2.03 × 10^7^, 1.20 × 10^7^, 9.97 × 10^6^, 8.76 × 10^6^ and 5.85 × 10^6^ CFU/mL, respectively. POBA, PHA and DIOP could slightly attract *R. solanacearum* 1–1 while PHBA, VA and PTA had no obvious effect on chemotaxis of *R. solanacearum* 1–1 (Fig. 1).

We next used RT-qPCR to determine the effects of PAs on the expression of *cheA.* The results showed that, DBP had the strongest promoting effect on the expression of *cheA* with a nearly 80-fold increase among all eleven PAs. In addition, BA and DIBP upregulated the expression level of *cheA* while the rest of PAs did not stimulate the expression of *cheA*.

### Evaluation of biofilm formation by R. solanacearum 1–1 in response to PAs

**Figure 2:**
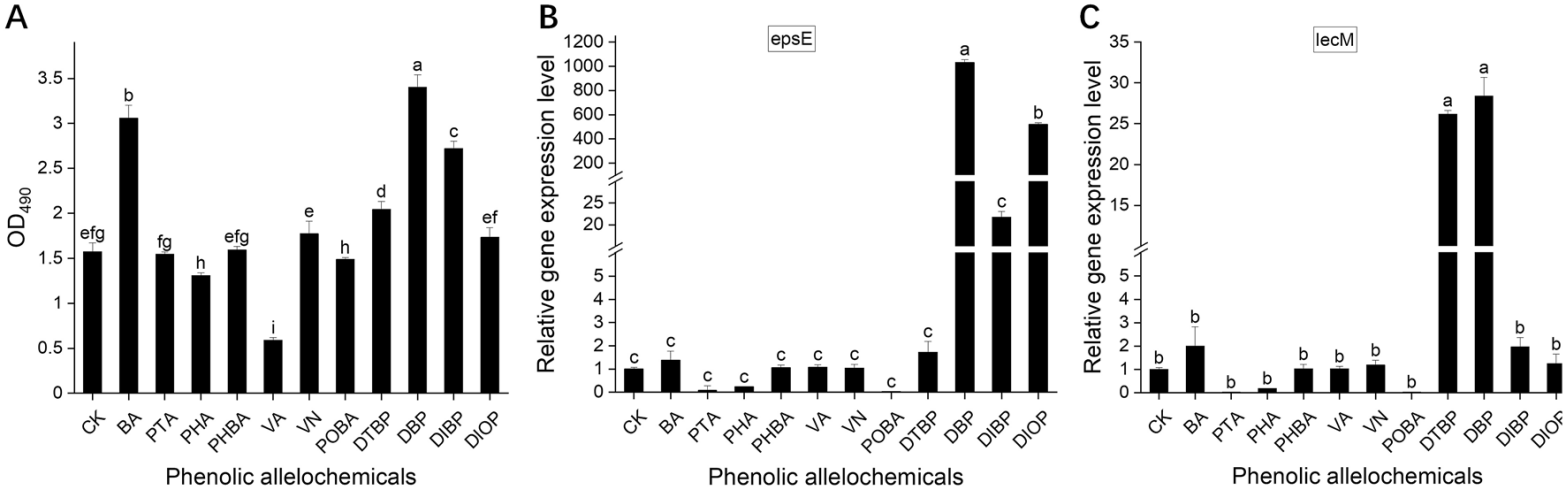
Effects of PAs on biofilm formation of *Ralstonia solanacearum* 1–1. (**A**), quantitative detection of biofilm of *Ralstonia solanacearum* 1–1 treated with PAs by crystal violet staining method; The colony numbers of *R. solanacearum* 1-1 under different PAs treatments were determined; (**C**), the relative expression level of *epsE* and *lecM* gene of *Ralstonia solanacearum* 1–1 in NB medium supplemented with PAs.

In order to investigate whether other virulence factors of *R. solanacearum* are also affected by the PAs, biofilm formation was tested with crystal violet staining method. It was found that DBP, DIBP, DIOP, BA, DTBP and VN increased biofilm formation by 10.19–116.6%, therein, DIBP, BA and DBP significantly enhanced the biofilm formation, with a biomass increase of 73.24%, 94.90% and 116.60% respectively compared with the control. The rest of PAs had no obvious stimulation effect on the biofilm formation of *R. solanacearum* 1–1, and some PAs even had reverse effect, such as VA, which markedly reduced the biofilm formation with the percentage inhibition of about 64.2% compared to the control (Fig. 2).

Furthermore, we evaluated the effects of PAs on the expression of *lecM* and *epsE* related to the biofilm formation of *R*. *solanacearum*. The mRNA levels of *epsE* remarkably increased 21.70-, 519.14- and 1031-fold after treating with DIBP, DIOP and DBP respectively. Besides, DTBP and DBP could upregulate *lecM* expression by 26.17- and 28.40-fold, respectively. BA, PHBA, VA and VN showed no significant effect on the expression of *epsE* and *lecM* while PTA, PHA and POBA showed downregulating effect on these two genes.

### Isolation, identification and characterization of PAs-degrading bacteria

**Figure 3:**
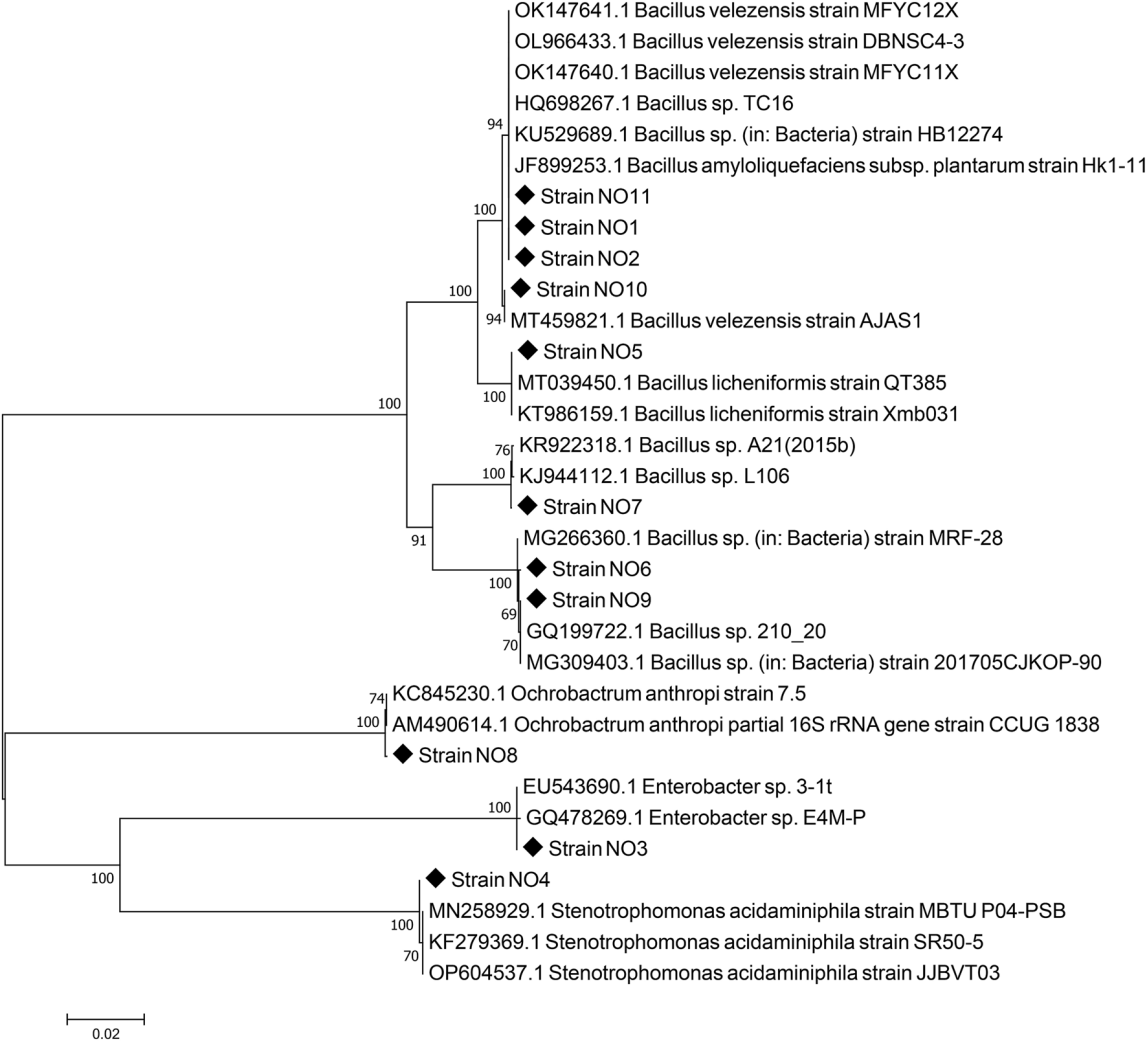
Neighbor-joining phylogenetic tree showing the position of eleven isolated PAs. Numbers at nodes indicate percentage levels of bootstrap support based on a neighbor-joining analysis of 1000 resampled datasets. The scale bar indicates 0.01 substitutions per nucleotide position.

The results above indicated that these eleven PAs had positive effects on *R*. *solanacearum* 1–1 through promoting growth, chemotaxis and biofilm formation, thus we isolated the corresponding bacteria to degrade them and ultimately mitigated the TBW. In this study, after three times of subculture in the screening medium containing PAs as sole carbon source and three times of colony purification on agar-solidified medium, eleven strains of microbes were isolated. Based on 16S rRNA sequencing, alignment and phylogenetic analysis, these strains affiliated to *Bacillus*, *Brucella*, *Enterobacter* and *Stenotrophomonas*, and named as *Bacillus* sp. NO1, *Bacillus* sp. NO2, *Enterobacter* sp. NO3, *Stenotrophomonas* sp. NO4, *Bacillus* sp. NO5, *Bacillus* sp. NO6, *Bacillus* sp. NO7, *Brucella* sp. NO8, *Bacillus* sp. NO9, *Bacillus* sp. NO10 and *Bacillus* sp. NO11 (Fig. 3), which could degrade BA, PTA, PHA, PHBA, VA, VN, POBA, DTBP, DBP, DIBP, and DIOP.

In order to clarify the degradation abilities of the screened strains to PAs, the degradation efficiency of the screened strains to eleven PAs was determined in mineral salt medium (MSM). As shown in Table 3, *Bacillus* sp. NO11, *Bacillus* sp. NO6, *Enterobacter* sp. NO3 and *Stenotrophomonas* sp. NO4 could only degrade PTA, DIOP, PHA and PHBA with the degradation rates of 100%, 77.92%, 67% and 40%, respectively.

**Table 3:** PAs-degrading bacteria and their degradation efficiencies in mineral salt medium (MSM).

PAs-degradation bacteria	Degradation efficiency (%)										
	BA	PTA	PHA	PHBA	VA	VN	POBA	DTBP	DBP	DIBP	DIOP
*Bacillus* sp. NO1	82.35	–	–	72.75	–	–	11.13	–	–	–	9.74
*Bacillus* sp. NO2	–	100.00	57.38	–	–	–	–	–	–	–	–
*Enterobacter* sp*.* NO3	–	–	67.00	–	–	–	–	–	–	–	–
*Stenotrophomonas* sp*.* NO4	–	–	–	40.00	–	–	–	–	–	–	–
*Bacillus* sp*.* NO5	–	–	–	–	50.13	–	–	–	–	64.61	–
*Bacillus* sp. NO6	–	–	–	–	–	77.92	–	–	–	–	–
*Bacillus* sp*.* NO7	–	–	–	–	–	–	48.00	–	–	–	4.25
*Brucella* sp. NO8	–	–	–	–	–	–	–	78.16	–	–	68.71
*Bacillus* sp. NO9	–	–	–	–	80.89	–	33.50	–	89.30	–	–
*Bacillus* sp. NO10	–	–	–	–	–	–	–	–	–	100.00	44.47
*Bacillus* sp. NO11	–	–	–	–	–	–	–	–	–	–	100.00

*“–”, no PA-degrading potential.

The rest strains could degrade two or more PAs. For instance, *Bacillus* sp. NO2 and *Bacillus* sp. NO10 completely degraded PTA (100%) and DIBP (100%) while the degradation rate dropped nearly half to PHA (57.38%) and DIOP (44.47%), respectively. *Bacillus* sp. NO5 had moderate degradation efficiency for VA (50.13%) and DIBP (64.61%), and *Brucella* sp. NO8 also could degrade two kinds of PAs with the above-average degradation rates of 68.71% (DIOP) and 78.16% (DTBP). *Bacillus* sp. NO7 showed the most potent degradation capacity for POBA (48%) among these eleven strains while could hardly degrade DIOP (4.25%). *Bacillus* sp. NO9 showed high degradation efficiencies for DBP and VA (over 80%) while with low degradation rate of 33.5% on POBA. *Bacillus* sp. NO1 had the broadest degradation spectrum that it could degrade DIOP (9.74%), POBA (11.13%), PHBA (72.75%) and BA (82.35%).

**Figure 4:**
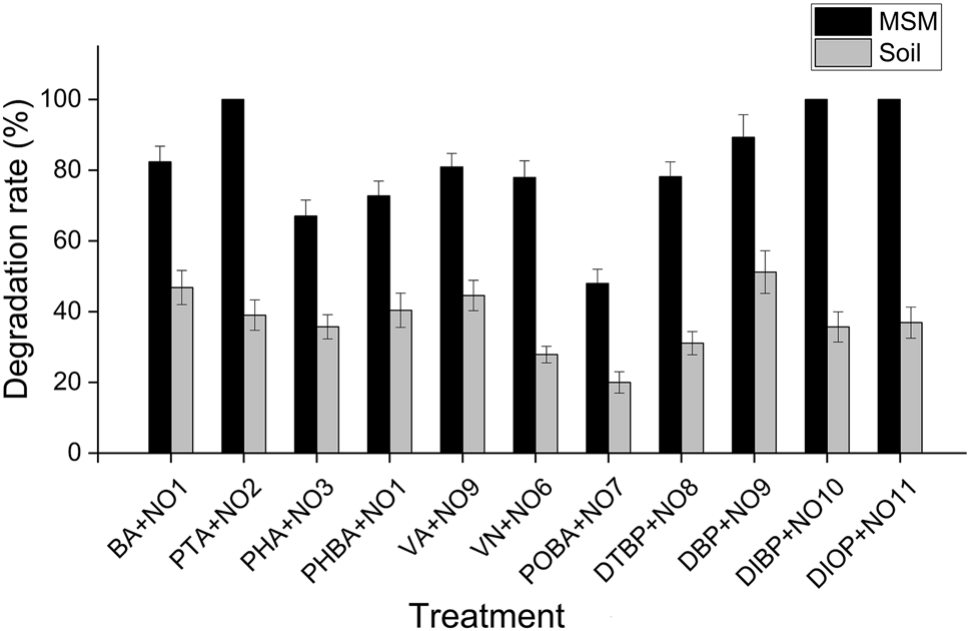
Degradation efficiency of PAs-degrading bacteria in MSM solutions and soil. B4, *Bacillus sp.* B4*;* D4 *Bacillus sp.* D4; ED2, *Bacillus sp.* ED2*;* NO3, *Enterobacter sp.* NO3; NO13, *Bacillus sp.* NO13; SD2, *Bacillus sp.* SD2; XQ4, *Bacillus sp.* XQ4; YX4, *Bacillus sp.* YX4; YZ1, *Bacillus sp.* YZ1.

In addition, we also tested the degradation effects of these strains on PAs under the soil condition. According to the degradation results of MSM, we found that *Bacillus* sp. NO1 screened out using BA as the sole carbon source showed much higher degradation rate of PHBA than that of *Stenotrophomonas* sp. NO4 isolated using PHBA as the sole carbon source. The same result was found on VA degradation, *Bacillus* sp. NO9 held stronger VA-degradation capacity than *Bacillus* sp. NO5, thus *Bacillus* sp. NO1 and *Bacillus* sp. NO9 were used to degrade PHBA and VA respectively in the pot experiment. The results showed that the degradation efficiencies of all the PAs-degrading strains in soil all dropped with the degradation rates from 20 to 51% compared with 48 to 100% in MSM (Fig. 4). *Bacillus* sp. NO1 and *Bacillus* sp. NO9 showed the least decline of degradation rates and kept relatively strong degradation capacity for BA (46.81%) and DBP (51.17%) respectively.

### Mitigation effects of single PA-degrading bacterium on TBW

**Figure 5:**
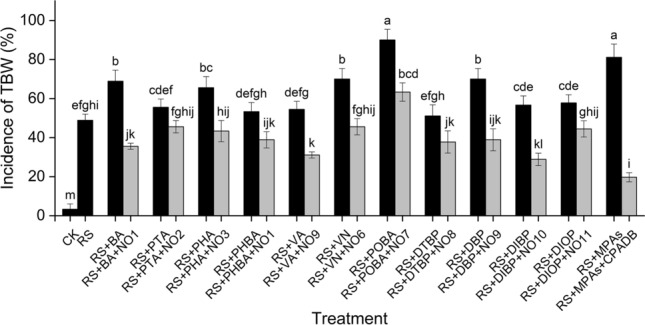
The incidence of TBW of various treatments. RS, *R. solanacearum* 1–1, MPAs, mixed phenolic allelochemicals; CPADB, combined phenolic allelochemicals-degrading bacteria; RS + BA, the CK group supplemented with *R. solanacearum* 1–1 and BA in the soil; RS + BA + NO1, the group of “RS + BA” adding *Bacillus* sp. NO1 in the soil. The same as below. All treatment groups were compared with the CK group for significant difference analysis.

In order to assess the practical value of these degradation strains on mitigating TBW, we firstly confirmed whether these degradation strains could inhibit the growth of *R. solanacearum* 1–1*.* The result showed that only *Bacillus* sp. NO1, *Bacillus* sp. NO9 and *Bacillus* sp. NO10 had antagonistic effect on *R. solanacearum* 1–1 (Table S2)*.* Next, the greenhouse pot experiment was employed to determine the effects of degradation strains on the incidence of TBW. As shown in Fig. 5, the disease incidence of TBW dramatically increased when inoculating *R. solanacearum* 1–1 to the tobacco roots (T1 group, 48.89%) compared with CK (3.33%). Adding both *R. solanacearum* 1–1 and each of PAs to the soil (T2 group) further enhanced the incidence of TBW, with the incidence of TBW ranged from 51.11 to 90%, especially the combination of *R. solanacearum* 1–1 and POBA, which significantly increased the incidence of TBW for nearly twofold (90%) compared with the T1 group*.* The declined incidences of TBW of all T2 groups were detected after adding the corresponding degrading strains to the tobacco roots (T3 group). For instance, the incidence of TBW in a T2 group containing *R. solanacearum* 1–1 and BA dropped from 68.89% to 35.56% after adding *Bacillus* sp. NO1 to the soil. The DBP-degrading strain *Bacillus* sp. NO9, PHA-degrading strain *Enterobacter* sp. NO3 and PTA-degrading strain *Bacillus* sp. NO2 decreased the incidence of TBW by 31.11%, 22.23% and 10% compared with T2 group respectively. It is worth noting that POBA-degrading strain *Bacillus* sp. NO7 reduced incidence of TBW by 26.67% compared with T2 group while the incidence of TBW increased by 14.44% compared with T1 group. This result was consistent with the finding above that *Bacillus* sp. NO7 had poor degradation efficiency on POBA which could stimulate the growth of *R*. *solanacearum* 1–1. In general, the incidences of TBW in T3 groups were 10 to 33.33% lower than that in T2 groups, and the lowest incidence of TBW was 32.22%.

### Mitigation effects of combined degrading bacteria on TBW

Firstly, we tested the antagonistic effect among these degrading strains. The results showed that PHA-degrading strain *Enterobacter* sp. NO3 had antagonistic effect on *Bacillus* sp. NO2, *Brucella* sp. NO8 and *Bacillus* sp. NO10 (Table S2), hence *Enterobacter* sp. NO3 was excluded and substituted by *Bacillus* sp. NO2, which could degrade PHA with the similar degradation rate of 57.38% and 32.36% in MSM and soil respectively compared with that of *Enterobacter* sp. NO3 (67% in MSM and 35.71% in soil). *Bacillus* sp. NO1 and *Bacillus* sp. NO9 showed much higher degradation efficiency of PHBA and VA than that of *Stenotrophomonas* sp. NO4 and *Bacillus* sp. NO5 respectively, thus *Stenotrophomonas* sp. NO4 and *Bacillus* sp. NO5 were excluded. *Bacillus* sp. NO1 and *Bacillus* sp. NO9 also could degrade POBA with the degradation rate of 11.13% and 33.5%, and their combined degradation efficacy was similar with that of *Bacillus* sp. NO7 (48%), thus *Bacillus* sp. NO7 was excluded and *Bacillus* sp. NO11 was also excluded with the same reason. Finally, six strains of *Bacillus* sp. NO1, *Bacillus* sp. NO2, *Brucella* sp. NO8, *Bacillus* sp. NO9, *Bacillus* sp. NO6 and *Bacillus* sp. NO10 were chosen, and five of them were *Bacillus*.

The six strains were balanced mixed and applied to mitigate TBW in pot experiment. In T2 group, *R. solanacearum* 1–1 and eleven PAs with the same concentration were added to the soil, the TBW incidence (81.11%) of which was much higher than that of T1 group (48.89%). However, the incidence of TBW sharply decreased to no more than 20% after adding these combined strains (T3 group, 19.37%), which was significantly lower than any other T3 group treated with single strain (Fig. 5), demonstrating that combined strains hold potent control capacity over TBW.

### Effects of combined degrading bacteria on tobacco growth and defense response

Meanwhile, we measured the agronomic traits and root vitality of tobacco under various treatments. The results showed that plant height (PH), stem girth (SG), length of the largest leaf (LL), width of the largest leaf (WL) and root vitality were all inhibited in T1 and T2 groups while rescued to normal levels in T3 group (Tables 4 and 5). In addition, all treatments had no obvious effect on the content of Chlb while the content of Chla significantly decreased in T2 group but restored in T3 group (Table 5), indicating that combined strains could promote photosynthesis. The results also showed that the combined degrading bacteria retrieved the sharply declined activities of peroxidase (POD) and superoxide dismutase (SOD) caused by *R. solanacearum* 1–1 and PAs to the normal level (Table 5). Moreover, the combined degrading bacteria remarkably increased the activity of SOD (277.14 U) compared with CK (203.34 U), suggesting they could suppress disease severity and occurrence through enhancing production of plant defense enzymes like SOD and POD.

**Table 4:** The effects of different treatments on agronomic traits of tobacco.

Treatment	PH (mm)	SG (mm)	LL (mm)	WL (mm)
CK	309.67 ± 11.60a	8.50 ± 1.26a	247.50 ± 10.10a	131.17 ± 6.84a
T1	241.5 ± 11.22b	6.17 ± 1.07b	206.67 ± 14.78b	105.33 ± 8.84b
T2	188.17 ± 14.05c	4.00 ± 0.82c	155.67 ± 7.18c	80.83 ± 7.01c
T3	305.67 ± 13.83a	7.67 ± 0.94a	238.67 ± 11.54a	126.67 ± 6.60a

* T1, the CK group inoculating *R. solanacearum* 1–1; T2, the T1 group adding mixed PAs; T3, the T2 group adding combined PAs-degrading bacteria; PH, plant height; SG, stem girth; LL, length of the largest leaf; WL, width of the largest leaf. Different letters indicate significant difference (*P* < 0.05) according to Duncan’s multiple range test.

**Table 5:** The effects of different treatments on root vitality, chlorophyll content and protective enzymes’ activities of tobacco.

Treatment	Root vitality (mg/(g•h))	Chla content (%)	Chlb content (%)	SOD activity (U)	POD activity (U)
CK	191.46 ± 10.75a	1.53 ± 0.07a	0.36 ± 0.04a	203.34 ± 14.67c	20.92 ± 2.09a
T1	97.57 ± 9.12b	1.43 ± 0.14ab	0.34 ± 0.05a	241.74 ± 9.76b	10.93 ± 1.51b
T2	78.96 ± 8.12b	1.30 ± 0.04b	0.37 ± 0.07a	238.52 ± 13.08b	12.01 ± 2.36b
T3	183.82 ± 10.36a	1.57 ± 0.1a	0.38 ± 0.05a	277.14 ± 11.66a	19.00 ± 2.02a

*T1, the CK group inoculating *R. solanacearum* 1–1; T2, the T1 group adding mixed PAs; T3, the T2 group adding combined PAs-degrading bacteria. Different letters indicate significant difference (*P* < 0.05) according to Duncan’s multiple range test.

## Discussion

Among major bacterial diseases, bacterial wilt caused by *R. solanacearum* is most devastating in tobacco. It has been reported that allelochemicals play important roles in promoting the incidence of TBW through rhizosphere ecology, nutrient acquisition, and plant–microbe interactions^19^. In the present study, eleven phenolic metabolites of BA, PTA, PHA, PHBA, VA, VN, POBA, DTBP, DBP, DIBP and DIOP were identified from continuous monoculture soil, and with the extension of continuous cropping year, the contents of these phenolic metabolites significantly increased (Table 1). Chen et al.^20^ reported the similar phenomenon that with the extending growth stage and planting year of eggplant, the contents of cinnamic acid and VA in soil increased.

Many studies have proven that these eleven phenolic metabolites are allelochemicals showing phytotoxic activities against plants^19,21–29^, and different concentrations of allelochemicals have different effects on microbial growth^30^. Our results revealed that more than half of the identified PAs promoted the growth of *R. solanacearum* 1–1 at low concentration while showed inhibition effects at high concentration (Table 2), this was consistent with the findings of Li^5^, who found that with the increasing concentration of BA and cinnamic acid, the cell densities of *R. solanacearum* first increased and then decreased. Besides, DIBP and DBP significantly promoted the growth of *R. solanacearum* 1–1 under all tested concentrations, indicating that they may serve as growth factors as well as carbon sources for *R. solanacearum*.

In addition to being a carbon source, allelochemicals can also act as chemical signals to induce the chemotactic effect and biofilm formation of *R. solanacearum* to initiate bacterial wilt^4^. Chemotaxis and biofilm formation play important roles in efficient bacterial colonization, infection and pathogenic fitness in rhizospheres^4^. Plants can secrete a lot of sugars, organic acids and secondary metabolites acting as chemotactic compounds to mediate plant–microbe interactions. For example, malic acid and citric acid are chemoattractants for *Pseudomonas fluorescen*s^31^. *R. solanacearum* has been reported to exhibit powerful chemotaxis to some organic acids, such as malic acid, citric acid, fumaric acid, and succinic acid from the root exudates of tobacco and tomato^4,32,33^. In this study, DBP, DTBP, DIBP, VN, BA had significant chemotaxis on *R. solanacearum* 1–1 (Fig. 1). Based on the results above that DBP, DTBP and DIBP promoted the growth of *R. solanacearum* 1–1 at all concentrations, and VN and BA showed positive effects on the growth of *R. solanacearum* 1–1 at low concentrations (Table 2), we inferred that DBP, DTBP, DIBP, VN and BA could be used as the nutrient carbon source of *R. solanacearum* 1–1 and as chemical attractants to stimulate the migration and root colonization of *R. solanacearum* 1–1. Yao et al.^32^ also found the similar result that citric acid and malic acid could be as the sole carbon source and as chemotactic factors for *R. solanacearum*. Biofilm formation can protect the pathogen from plant defenses, abiotic stress and external control measures and thus improving the virulence of the phytopathogen^34^. Our results showed that BA, DTBP, DBP and DIBP not only significantly attracted but also promoted the biofilm formation of *R. solanacearum* 1–1 (Figs. 1 and 2). Li et al.^5^ reported the similar findings that PHA and BA could significantly promote the biofilm formation of *R. solanacearum* and had a slight attractive effect toward *R. solanacearum*.

The histidine protein kinase CheA involved in flagellar rotation is essential for the chemotaxis and virulence of many pathogens such as *E*. *coli* and *R*. *solanacearum*^35^. Mutations of *cheA* resulted in a completely non-chemotactic phenotype both in *E*. *coli* and *R*. *solanacearum*^32,36^. Moreover, *cheA* mutant *R*. *solanacearum* had significantly reduced virulence distinguishable from that of the wild-type^32^. Our results showed that BA, DBP and DIBP significantly upregulated the expression level of *cheA*, which was consistent with the results of chemotactic assay (Fig. 1). Li et al*.*^5^ also reported BA, cinnamic acid and myristic acid markedly induced the expression of *cheA* and thus enhancing the attractiveness of these compounds to microorganisms.

In type III secretion system (T3SS), *lecM* and *epsE* are key genes involved and regulated in biofilm formation. The absence of *lecM* or *epsE* severely impaired the biofilm formation of *R*. *solanacearum* and resulted in the loss of virulence on plants^37^. In our study, DBP, DIBP and DIOP induced the expression of *epsE*, DBP and DTBP promoted the expression of *epsE*, all of which can promote the formation of biofilms (Fig. 2). Similarly, Li^38^ found that BA enhanced both the expression of *lecM* and *epsE* while PHA exhibited the opposite effect at the concentration of 100–200 μM.

Researches on the microbial degradation of allelochemicals have been carried out for a long time. BA, PTA, PHA, PHBA, VA, VN and DBP can be degraded by *Pseudomonas*, *Pseudomonas putida*, *Sphingomonas capsulate*, *Acinetobacter johnsonii* FZ-5, *Klebsiella oxytoca* FZ-8, *Providencia* sp. 2D, *Basidiomycete* yeast sp., *Bacillus thuringiensis* and *Gordonia* sp. strain MTCC 4818^18,39–45^. In this study, eleven bacteria, including eight strains of *Bacillus*, one strain of *Brucella,* one strain of *Enterobacter* and one strain of *Stenotrophomonas*, were isolated to degrade these eleven PAs (Fig. 3). For the past few years, *Bacillus* have been widely used to control of diverse plant pathogens, such as bacterial wilt, Fusarium wilt, downy mildew, soft rot, rice blast and sheath blight^46^. *Bacillus* can also be used as plant growth promoting rhizobacteria (PGPR) through degrading organic compounds to release plant nutrients, improve root vitality, and optimize root architecture^47^. What’s more, *Bacillus* produces resting spores during its growth and development, which is easy to be stored and transported as stable products^48,49^.

In MSM medium, most PAs could be effectively degraded by PAs-degrading bacteria, and PTA, DIBP and DIOP could be completely degraded by *Bacillus* sp. NO2, *Bacillus* sp. NO10 and *Bacillus* sp. NO1 respectively. However, the degradation efficiencies of PAs all dropped in the soil conditions with the degradation rates from 20 to 51% compared with 48 to 100% in MSM (Fig. 4 and Table 3). These results were in disagreement with the findings of Wang et al*.* who revealed that PHA and PHBA were almost completely degraded by *P. putida* strain 7 and *P*. *hunanensis* strain 10 in soil. The main reason may be the initial additive amounts of PAs were much higher than that of reported by Wang et al*.*^3^, which was 600 μg/g in our study while 35 μg/g and 250 μg/g in Wang’s study. Excessive PAs needed more degrading bacteria or took longer to be degraded. Another reason may be the living environment for degrading bacteria in soil, including nutrients, pH, temperature and humidity are different and more severe than those in the culture solutions, leading to the decline of reproductive ability and colonization ability of degrading bacteria during the growth of tobacco.

In continuous monoculture cropping mode, various kinds of PAs were accumulated in the soil^9^. The results above indicated that every PA could promote the incidence of TBW, thus degrading one kind of PAs did not prevent the other PAs from contributing to the incidence of TBW. Therefore, it is necessary to degrade all these PAs with degradation strains in combination. Based on single-strain degradation experiment, six strains either having broad degradation spectrum or high degradation rate or resistance to *R. solanacearum* were combined to degrade all eleven PAs. Although the TBW incidence was sharply reduced by 61.74% in T3 group (19.37%) compared with T2 group (81.11%), the TBW was not thoroughly controlled (Fig. 5). It may be caused by the inadequate degradation of PAs in soil or the simple combination degradation mode. Another reason may lie in that TBW is caused by many factors including climatic conditions, physicochemical properties of soil and the structure and physiology of the rhizosphere microbial community, hence degradation of PAs alone is not enough to completely wipe out TBW^50^. Next, we will optimize the ratio among the six strains and develop additives, such as bacteria carrier, humectant, dispersant and stabilizer, to help growth and colonization of degrading strains to tobacco roots. Moreover, our degradation methods can be combined with other soil remediation techniques and cropping methods to achieve complete control of TBW.

At last, we measured whether the combined degrading bacteria had adverse effects on tobacco plant. Our results demonstrated that the combined degrading bacteria had no negative effects on tobacco growth, the root reducing activity, chlorophyll content, nor the POD and SOD activity, and even restored the decrease of these indexes caused by *R. solanacearum* 1–1 and PAs to the normal level (Tables 4 and 5). These results were in accordance with the reports that *Acinetobacter calcoaceticus* capable of degrading BA and DBP increased the content of chlorophyll in tomato^51^ and *Bacillus amyloliquefaciens* QSB-6 promoted significant increases in plant root respiration rate and protective enzyme activities (SOD, POD) under *Fusarium* invasion^46^.

Above all, the combined degrading bacteria not only remarkably mitigated the incidence of TBW but also promoted the plant growth and defense response. Therefore, it’s a safe and effective way to control TBW by degradation of PAs with combined degrading bacteria.

## Conclusion

Our results demonstrate that the incidence of TBW can be effectively reduced by degrading the accumulated PAs in soil with degrading microbes. Moreover, six strains of PA-degrading bacteria in combination can significantly decompose PAs and ultimately sharply mitigate the incidence of TBW in pot experiment. It’s the first report to degrade as many as eleven PAs at the same time with six degrading strains, and our method provides a promising strategy for TBW field control.

## Materials and methods

### Pathogen strains and chemicals

*R. solanacearum* 1–1 was used as the pathogen in this study, which was provided by Hubei Tobacco Research Institute. All the experiments of bacterial culture were carried out in a biological flow hood (Suzhou Antai Airtech Co., Ltd, China). Phthalic acid (PHA), p-hydroxybenzoic acid (PHBA), benzoic acid (BA), vanillic acid (VA), p-hydroxybenzaldehyde (POBA), vanillin (VN), diisobutyl phthalate (DIBP), 2,4-Di-tert-butylphenol (DTBP), di-n-butyl phthalate (DBP), diisooctyl phthalate (DIOP) and terephthalic acid (PTA) were purchased from Yuanye company (Shanghai, China).

### Identification and quantification of rhizosphere soil PAs

Soil samples were taken from a sub-healthy crop zone in a TBW prone area in Xuanen County (109° 26′ 20″ E, 29° 59′ 55″ N), Enshi City, Hubei province, China. Continuous cropping soils were collected from the field where only tobacco was planted for 0 year (CCS0), 1 year (CCS1), 2 years (CCS2), 4 years (CCS4) and 10 years (CCS10) by five-spot-sampling method. Then the soil samples from the five separate sites were mixed to one soil sample. The soil (1 g) was ground and extracted in 5 mL 80% (v/v) methanol (10 min, 20 °C) thrice by using sonicator (Ningbo Scientz Biotechnology Inc., China). The supernatant was dried in a vacuum concentrator (Thermo Fisher Scientific Inc., USA) without heating. The dried extracts were pretreated and loaded on Agilent 7890 gas chromatograph system (Agilent Technologies Inc., USA) coupled with a Pegasus HT time-of-flight mass spectrometer (LECO Corporation, USA) for GC-TOF–MS analysis as described before^52^. Peak integration was performed using Chroma TOF 4.3X software (LECO Corporation, USA) and the LECO-Fiehn Rtx5 database. Both of mass spectrum match and retention index match were considered in metabolites identification.

### Evaluation of R. solanacearum 1–1 growth rate in PAs

The growth of *R. solanacearum* 1–1 under different PAs was measured based on Li’s method with minor modification^5^. Briefly, *R. solanacearum* 1–1 was grown in Nutrient broth (NB, beef extract 3.0 g/L, peptone 10 g/L, NaCl 5 g/L pH 7.4–7.6) overnight and the cells (OD_600_ = 1.0) were centrifuged, washed twice with phosphate buffer (100 mM, pH 7.0), and then resuspended in NB (OD_600_ = 1.0). Subsequently, the investigated PAs were added to the medium with the concentration of 0, 0.1, 0.5, 1, 2 and 4 mg/L. After cultivated for 12 h at 30 °C, the OD_600_ value of *R. solanacearum* 1–1 was determined with a spectrophotometer.

### Chemotaxis assay

Quantitative measurement of the chemotaxis of *R. solanacearum* 1–1 response to the PAs was performed based on Zhang’s procedure with minor modification^53^. Briefly, *R. solanacearum* 1–1 was grown in NB until reaching log phase (OD_600_ = 0.8). The cells collected by centrifugation were washed twice with phosphate buffer (100 mM, pH 7.0) and resuspended in the same buffer (OD_600_ = 0.8). Petri dish with a diameter of 60 mm was filled with 10 ml of the cell suspension prepared above. Standard 1-μL capillaries loaded with the rhizosphere PAs (100 μM) were perpendicularly immersed in the cell suspension. After 40 min of static incubation at room temperature, the suspension in the capillary was diluted and plated on NB solid plates containing 2,3,5-Triphenyltetrazolium chloride at 37 °C. The CFU (mL^−1^) was determined 18 h later. Phosphate buffer was used as negative control.

### Biofilm formation assay

The *R. solanacearum* 1–1 was cultured in NB until OD_600_ reached 1.0. A 10 μL aliquots of cell cultures was inoculated into 96-well cell culture plate filled with 100 μL NB which contained different PAs (100 μM). After static incubation at 30 °C for 12 h, the growth medium and non-adherent cells were removed from the plate wells, which were washed twice with sterile water. Biofilm cells were stained with 100 μL of 1% crystal violet (CV) for 30 min at room temperature. Subsequently, excess CV was poured out and the wells were washed five times with sterile water and the stain was dissolved with 100 μL of 33% glacial acetic acid solution in a 37 °C incubator for 30 min. Biofilm formation was quantified by measuring the OD_490_ by a microplate reader (Spectra Max iD3).

### Gene expression analysis

Quantitative detection of the expression of genes related to chemotaxis and biofilm formation was performed according to the methods reported by Li^38^. Briefly, a 10 μL aliquots of *R. solanacearum* 1–1 (OD_600_ = 1.0) was inoculated into 48-well cell culture plate filled with 1 mL NB which contained different PAs (100 μM). After static incubation at 30 °C for 24 h, the bacteria were collected and the total bacteria RNA were extracted with EasyPure® RNA Purification Kit (TransGen Biotech, Beijing, China) following the manufacturer’s protocol. Reverse transcription quantitative PCR (RT-qPCR) performed with PerfectStart® Uni RT&qPCR Kit (TransGen Biotech, China) was used to evaluate the transcription levels of *epsE*, *lecM* and *cheA*. *SerC* was used as a reference gene and the primers involved in the experiment were listed in Table S1. The 2^−ΔΔCT^ method was used to analyze the RT-qPCR data.

### Screening and identification of PAs-degrading bacteria

Soil samples were collected from dump, sewage outfalls and lake in Hubei University. Ten grams of soil were transferred into a sterile Erlenmeyer flask containing 90 mL sterilized phosphate buffer (10 mM, pH 6.5). After shaking for 4 h, 10 mL of suspensions were transferred to mineral salt medium (MSM: K_2_HPO_4_ 5.8 g/L, (NH_4_)_2_SO_4_ 2.0 g/L, KH_2_PO_4_ 4.5 g/L, CaCl_2_ 0.02 g/L, MgCl_2_ 0.16 g/L, FeCl_3_ 0.0018 g/L, Na_2_MoO_4_·2H_2_O 0.0024 g/L, MnCl_2_·2H_2_O 0.0015 g/L, pH 7.0, 100 mL) containing only one kind of PAs (100 mg/L) and incubated at 28 °C for 5 d. Repeat this step for three times and the last suspension was spread onto MSM agar plates containing corresponding PA (100 mg/L). The emerged single colonies were further purified by streaking on LB agar (tryptone 10 g/L, yeast extract 5 g/L, NaCl 10 g/L, agar 15 g/L, pH 7.0) plates for three times.

For bacteria identification, the primers 27F and 1492R were used to amplify the 16S rRNA gene from bacteria genome extracted with EasyPure® Bacteria Genomic DNA Kit (TransGen Biotech, Beijing, China). The amplification procedure was described as Zhang, et al.^54^. PCR products were sequenced by Sangon Biotech Ltd., Shanghai, China. The sequences were compared with the 16S rRNA sequences in the NCBI database using the BLAST program. Phylogenetic and molecular evolutionary analyses were also conducted with MEGA 5.05 using the neighbour-joining method with the maximum composite likelihood distance measure^55^. Confidence values were estimated from bootstrap analysis of 1000 replicates. All sequencing reads obtained in this study have been deposited in the GenBank database under accession numbers OM349625-OM349635.

### PAs-degradation assay

The PAs-degradation efficiencies were determined both in MSM and soil. Active bacterial isolates were grown in LB medium until reaching log phase (OD_600_ = 0.8). The cells collected by centrifugation were washed thrice with phosphate buffer (100 mM, pH 7.0) and resuspended in eleven kinds of MSM, each containing only one kind of PA (100 mg/L). After two days of incubation, each suspension was extracted three times with an equal volume of ethyl acetate. The extracted phases were concentrated under reduced pressure and applied to HPLC for qualitative and quantitative analysis as described by Zhang et al.^56^. Degradation efficiency was calculated using the formula: $D = (I - R)/I \times 100$. Where D: % degradation, I: the initial concentration of PA, R: the remaining concentration of PA.

To determine the degradation effects of screened bacteria on PAs in soil condition, greenhouse pot experiment was carried out as below and the degradation efficiencies were measured after inoculating degrading bacteria for 30 d with the formula above.

### Antagonistic effect assay

The antagonistic effects of eleven PAs-degradation strains against *R. solanacearum* 1–1 and the antagonism among eleven PAs-degradation strains were determined by plate confrontation method. Two or three holes were drilled in a LB agar plate spread with *R. solanacearum* 1–1 or one strain of PA-degrading bacteria, and 20 μL of another strain of PA-degrading bacteria was added to each hole. The plate was put in an incubator at 28 °C (in the dark) for 12 h until the colonies’ margins met in the area of interaction to observe antagonistic activity.

### Greenhouse pot experiment

The greenhouse pot experiment was conducted at Hubei University. Non-continuous cropping healthy sterilized soil was chosen for the following experiments. PAs-degradation strains and *R*. *solanacearum* 1–1 were cultured in LB medium and NB respectively. Tobacco seeds of Yunyan87 were provided by the Tobacco Research Institute of Hubei, Wuhan, China. The seeds were grown in floating polystyrene trays in a greenhouse for approximately 60 d before being transplanted to the pots. Four treatments were established after transplanting for 7 d: (1) the control group (CK, without any treatment); (2) the CK group inoculating *R. solanacearum* 1–1 (T1); (3) the CK group adding both *R. solanacearum* 1–1 and PAs (T2); (4) the CK group adding both *R. solanacearum* 1–1 and PAs, and inoculating PAs-degradation strains the next day (T3). The PAs-degrading bacteria, *R. solanacearum* 1–1 and PAs were irrigated into the tobacco roots. The dosage of both *R. solanacearum* 1–1 and PAs- degrading bacteria was 6 mL (1.00 × 10^8^ CFU/mL) per pot, the addition of PAs was 11 mL (600 mg/L) per pot. For combined degradation of PAs, the dosage of each chosen PAs-degrading bacterium was 1 mL (1.00 × 10^8^ CFU/mL) per pot and the addition of each PA was 1 mL (600 mg/L) per pot. A randomized complete block arrangement was adopted to lay out each of the four treatments with 20 pots (one plant per pot) and three replicates. After transplanting for 30 d, the incidence of TBW disease was calculated with the formula: $I = D/T \times 100$. Where I: % incidence of TBW, D: the number of plants with disease, T: the total number of plants.

### Measurement of agronomic traits, root vitality, chlorophyll content and leaf SOD and POD activities of tobacco

Agronomic traits. Four agronomic traits, including plant height (PH), stem girth (SG), length of the largest leaf (LL) and width of the largest leaf (WL) were measured after transplanting for 30 d.

Root vitality. Tobacco root tips were collected after transplanting for 15 d and the root reductive intensity was measured with TTC (triphenyl tetrazolium chloride) reducing method as described by Zhang^57^.

Chlorophyll content. Fresh leaves of 2 g were extracted with 10 mL of alcohol. The absorption of the extracts was measured at 663 and 645 nm. The contents of chlorophyll-a/b (Chla/b) were determined as described by Lichtenthaler^58^.

Leaf SOD and POD activities. Superoxide dismutase (SOD) activity and peroxidase (POD) activity were measured according to the method of Duan^46^.

### Statistical analysis

All data were subjected to analyses of variance (ANOVA) using SPSS 22.0 (SPSS Inc., Chicago, IL, USA). The least significant difference (LSD) was used to determine significant differences at the levels of *P* < 0.05 between treatment means. All the experiments were repeated at least three times.

### Ethics approval

No use of human or animal samples or tissues in this study. The tobacco used in this study complied with all local, national or international guidelines and legislation.

## Supplementary Information

Supplementary Information 1.

Supplementary Information 2.

## Data Availability

All sequencing reads obtained in this study have been deposited in the GenBank database (www.ncbi.nlm.nih.gov/genbank/) under accession numbers OM349625-OM349635. The other data generated or analyzed during the current study are included in this article.
