# Supplementary material for: Mitigation of tobacco bacteria wilt with microbial degradation of phenolic allelochemicals
Source: Sci Rep. 2022 Dec 1;12:20716. doi: 10.1038/s41598-022-25142-0 (PMC9715567; doi:10.1038/s41598-022-25142-0)
Supplement: Supplementary file 1 — Supplementary Information 1. [file 41598_2022_25142_MOESM1_ESM.docx]

Table S1 PCR primers in this study

| Geene | Primer |
| --- | --- |
| SerC_F | ACATAGATGGCGTAGGT |
| SerC_R | GTCGACTGGAACG GCTAT |
| cheA_F | TGAACGCCATCTTCCGTT |
| cheA_R | CTGAACGCCATCTTCCGTTG |
| lecM_F | CTCAGCAAGGTGTATTCA |
| lecM_R | GTTGTAGTCGTTGTCGGT |
| epsE_F | AGTGGTACATCGCCATCA |
| epsE_R | ATAAAGCCACGCAAAGAT |
