# Supplementary material for: Mitigation of tobacco bacteria wilt with microbial degradation of phenolic allelochemicals
Source: Sci Rep. 2022 Dec 1;12:20716. doi: 10.1038/s41598-022-25142-0 (PMC9715567; doi:10.1038/s41598-022-25142-0)
Supplement: Supplementary file 2 — Supplementary Information 2. [file 41598_2022_25142_MOESM2_ESM.docx]

Table S2 Antagonistic effects among bacteria

| Strains | *Bacillus* sp. NO1 | *Bacillus* sp. NO2 | *Enterobacter* sp*.* NO3 | *Stenotrophomonas* sp*.* NO4 | *Bacillus* sp*.* NO5 | *Bacillus* sp. NO6 | *Bacillus* sp*.* NO7 | *Brucella* sp. NO8 | *Bacillus* sp. NO9 | *Bacillus* sp. NO10 | *Bacillus* sp. NO11 | *R. solanacearum* |
| --- | --- | --- | --- | --- | --- | --- | --- | --- | --- | --- | --- | --- |
| *Bacillus* sp. NO1 | - | - | - | - | - | - | - | - | - | - | - | + |
| *Bacillus* sp. NO2 | - | - | + | - | - | - | - | - | - | - | - | - |
| *Enterobacter* sp*.* NO3 | - | + | - | - | - | - | - | + | - | + | - | - |
| *Stenotrophomonas* sp*.* NO4 | - | - | - | - | - | - | - | - | - | - | - | - |
| *Bacillus* sp*.* NO5 | - | - | - | - | - | - | - | - | - | - | - | - |
| *Bacillus* sp. NO6 | - | - | - | - | - | - | - | - | - | - | - | - |
| *Bacillus* sp*.* NO7 | - | - | - | - | - | - | - | - | - | - | - | - |
| *Brucella* sp. NO8 | - | - | + | - | - | - | - | - | - | - | - | - |
| *Bacillus* sp. NO9 | - | - | - | - | - | - | - | - | - | - | - | + |
| *Bacillus* sp. NO10 | - | - | + | - | - | - | - | - | - | - | - | + |
| *Bacillus* sp. NO11 | - | - | - | - | - | - | - | - | - | - | - | - |
| *R. solanacearum* | + | - | - | - | - | - | - | - | + | + | - | - |

* “+”, means antagonistic effect, “-” means non-antagonistic effect.
